# Clinical Utility of Presacral Neurectomy as an Adjunct to Conservative Endometriosis Surgery: Systematic Review and Meta-Analysis of Controlled Studies

**DOI:** 10.1038/s41598-020-63966-w

**Published:** 2020-04-23

**Authors:** Larry E. Miller, Ruemon Bhattacharyya, Valerie M. Miller

**Affiliations:** Miller Scientific, Johnson City, TN United States

**Keywords:** Reproductive biology, Sexual dysfunction, Reproductive signs and symptoms

## Abstract

The objective of this review was to compare the efficacy and safety of conservative surgery with or without adjunctive presacral neurectomy (PN) for chronic endometriosis-related pelvic pain. In a systematic review with meta-analysis, randomized or nonrandomized controlled studies of conservative endometriosis surgery with or without adjunctive PN were included. Main outcomes were treatment failure (the proportion of women in which surgery failed to adequately resolve midline pain) and the frequency of operative and postoperative complications. A total of 7 studies with 8 group comparisons (3 randomized) representing 503 women (250 PN; 253 Control) were included. Over 34 months median follow-up, crude rates of treatment failure were 15.0% with PN and 40.9% with Controls (risk ratio = 0.43, 95% CI = 0.30 to 0.60, p < 0.001). The risk of postoperative constipation was higher with PN vs. Controls (12.5% vs. 0%, p = 0.024). No treatment group differences were observed for the risk of operative complications (0.6% vs. 0%, p = 0.498), reoperation (4.1% vs. 3.0%, p = 0.758) or urinary incontinence (5.0% vs. 0%, p = 0.195). Overall, in well-selected patients, conservative surgery with adjunctive PN may provide greater relief from midline pain and a similarly low rate of operative complications relative to conservative surgery alone but may increase the risk of constipation postoperatively. However, results were derived from mainly older and lower quality studies. Since then, surgical techniques to treat endometriosis have been improved and the effect of PN observed in prior studies should be confirmed in future studies in women in whom radical excision of deep infiltrating lesions is obtained.

## Introduction

Endometriosis affects up to 10% of women of reproductive age and is associated with chronic pelvic pain and dysmenorrhea^1^. Medical management consisting of nonsteroidal anti-inflammatory drugs or hormone-based therapies are first-line treatments for chronic pelvic pain secondary to endometriosis. Despite providing adequate symptom relief in most patients, some women gain only limited or intermittent benefit from medical treatment^2,3^. Consequently, conservative surgery may be indicated in women with inadequate symptom resolution with medical therapy and who wish to preserve fertility. Pelvic denervation procedures may be used as an adjunct to surgical excision or ablation of endometrial lesions. Presacral neurectomy (PN) involves complete transection of the presacral nerves innervating the uterus, cervix, and proximal fallopian tubes, which is hypothesized to provide relief of chronic midline pelvic pain. The incremental benefit of adjunctive PN to conservative surgery for endometriosis has been explored in previous systematic reviews, but conclusions were mixed^4,5^. Since these reviews were published over 10 years ago, a reappraisal of existing evidence is warranted. The purpose of this systematic review with meta-analysis was to compare the efficacy and safety of conservative surgery with or without adjunctive PN for relief of chronic pelvic pain due to endometriosis.

## Material and Methods

### Data sources and searches

We developed and followed a review protocol that adhered to the Preferred Reporting Items for Systematic Reviews and Meta-analyses (PRISMA)^6^ and was registered in the International Prospective Register of Systematic Reviews (PROSPERO) public database (CRD42019120488; http://www.crd.york.ac.uk/PROSPERO). We searched Medline, Embase, Cochrane Central Register of Controlled Trials, and the Directory of Open Access Journals, with no date or language restrictions, from inception to December 31, 2018 for randomized and nonrandomized controlled studies of conservative endometriosis surgery with or without adjunctive PN using a combination of diagnosis- and treatment-specific keywords. The syntax for Medline searches is provided in Table 1; the syntax for other databases was similar but adapted as necessary. Reference lists of included papers and relevant meta-analyses were manually searched. We also reviewed reports from abstracts and presentations at major gynecological meetings to reduce the risk of publication bias^7^.

**Table 1:** MEDLINE Search Strategy to Identify Controlled Studies of Conservative Surgery With or Without Presacral Neurectomy*.

**Diagnosis Search Terms**
1. Dysmenorrhea
2. Endometriosis
3. Midline
4. Menstruation
5. Pelvic pain
**Treatment Search terms**
6. Laparoscop*
7. Presacral neurectomy
8. Denervation
9. Surgery
**Combination Terms**
10. or/1–5
11. or/6–9
12. and/10–11

*An asterisk represents a wildcard symbol used in a search query to represent end truncation.

### Study selection

Two independent researchers (LM, DF) reviewed titles and abstracts for possible inclusion in the review. Study selection discrepancies between the reviewers were resolved by consensus. Titles and abstracts were initially screened to exclude review articles, commentaries, letters, case reports, and obvious irrelevant studies. Full texts of the remaining articles were retrieved and reviewed. Main inclusion criteria were controlled study of conservative endometriosis surgery with or without adjunctive PN; otherwise identical treatment and follow-up conditions in each group; and at least one reported outcome. Manuscripts published in non-English journals were rewritten in English by a translation service.

### Data extraction

An initial database was developed, pilot-tested, and refined to ensure consistency with outcomes reported in the literature. Data were independently extracted from eligible studies by two researchers (LM, DF). Data extraction discrepancies between the two researchers were resolved by discussion and consensus. The following variables were recorded in standardized data extraction forms: general manuscript information, patient characteristics (age, disease severity, symptom duration), study characteristics (study design, sample size, surgical access technique, follow-up duration), risk of bias using the Newcastle-Ottawa scale^8^, pain severity, procedural data (procedure time, procedural blood loss, hospital stay), operative complications, and postoperative complications.

### Outcomes

The primary endpoint of this systematic review was treatment failure, defined as the proportion of women in which surgery failed to adequately resolve midline pain, evidenced by continuation or recurrence of moderate or severe pain during follow-up. Secondary outcomes were frequency of operative complications, and postoperative complications including constipation, urinary incontinence, and reoperation.

### Data analysis

Women treated with conservative surgery and adjunctive PN were compared to those receiving conservative surgery alone (Controls) with the risk ratio (RR) statistic where a RR > 1 indicated higher risk with PN and a RR < 1 indicated lower risk with PN. For each outcome, the pooled estimate and 95% confidence interval (CI) were calculated. Forest plots were used to illustrate individual study findings and pooled meta-analysis results, when applicable. We used the I^2^ statistic to estimate heterogeneity of outcomes among studies; a value of 0% represents no heterogeneity and larger values represent increasing heterogeneity^9^. Significant heterogeneity was defined by a Cochran *Q* test p < 0.1 or I^2^ > 50%. When significant heterogeneity was identified, a random effects model was planned; otherwise, a fixed effect model was planned^10^. Publication bias was visually assessed with funnel plots and quantitatively assessed with Harbord’s test^11^. A priori, we identified several sensitivity analyses of the primary endpoint, including a subgroup analysis comparing outcomes in randomized vs. nonrandomized studies, meta-regression to explore the influence of follow-up duration on outcomes, and a one-study removed analysis in which we iteratively removed one study at a time to determine whether conclusions were significantly influenced by any single study. We also performed a post hoc analysis comparing primary endpoint results with a fixed effect vs a random effects model. P-values were two-sided with a significance level less than 0.05. Analyses were performed using Stata v14.2 (StataCorp LLC) and Comprehensive Meta-analysis v3.3, (Biostat).

## Results

**Figure 1:**
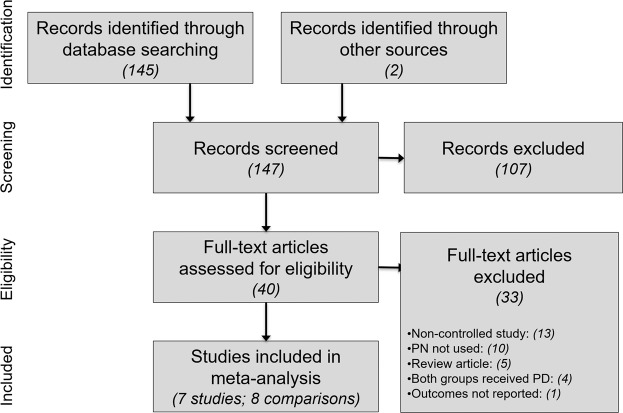
PRISMA flow diagram. PD = pelvic denervation; PN = presacral neurectomy.

Our initial database search retrieved 145 titles and abstracts; hand searching relevant bibliographies identified 2 additional records. After screening records for inclusion criteria, 40 full-text articles were reviewed for eligibility. Ultimately, 7 studies with 8 group comparisons representing 503 women (250 PN; 253 Control) were included in the meta-analysis. A flow diagram of study identification and selection is shown in Fig. 1.

Women were typically in their early 30 s with moderate or severe (American Fertility Society stage III or IV) endometriosis. Three of eight comparisons were derived from randomly allocated treatment groups, five of eight involved prospective recruitment, and follow-up duration ranged from 13 to 48 months (Table 2). Primary risks of bias among included studies were nonrandomized group assignment and retrospective recruitment. Overall, the risk of bias was high for two comparisons, intermediate for one comparison, and low for five comparisons (Table 3). Additional potential sources of bias related to patient selection criteria, procedural details, and primary outcome definitions are provided in Table 4, which varied considerably among studies.

**Table 2:** Patient and Study Characteristics in Controlled Studies of Conservative Surgery With or Without Presacral Neurectomy.

Study	Prospective Enrollment	Random Group Allocation	Surgical Access	No. Sites	No. Patients*	Mean Age (yr)*	Moderate or Severe Disease*	Symptom Duration*	Follow-up Duration (mo)
Candiani, 1992^19^	Yes	Yes	LPT	1	38, 40	33, 31	100%, 100%	†	36
Garcia, 1977^22^	No	No	LPT	1	35, 36	[29, 29]	[96%, 96%]	[3], [3]	24
Liu, 2011^16^	Yes	No	LPS	1	30, 34	37, 36	60%, 65%	6, 6	13
Polan, 1980^23^	No	No	LPT	1	8, 19	†	†	†	36
Puolakka, 1980^24^	No	No	LPS/LPT	1	51, 45	[35, 35]	†	†	31
Tjaden, 1990a^20^	Yes	Yes	LPT	1	4, 4	[30, 30]	100%, 100%	†	42
Tjaden, 1990b^20^	Yes	No	LPT	1	13, 5	[30, 30]	100%, 100%	†	42
Zullo, 2003/2004^12,13^	Yes	Yes	LPS	1	71, 70	32, 30	38%, 35%	≥6, ≥6	24

LPS = laparoscopy; LPT = laparotomy.

Brackets represent estimated values.

*Data reported as presacral neurectomy group, control group.

^†^Data not reported.

**Table 3:** Risk of Bias Assessment with Newcastle-Ottawa Scale in Controlled Studies of Conservative Surgery With or Without Presacral Neurectomy.

Study	Selection (4)	Comparability (2)	Outcome (3)	No. Stars (9)	Risk of Bias
Candiani, 1992^19^	★★★★	★★	★★★	9	Low
Garcia, 1977^22^	★	★	★★★	5	Intermediate
Liu, 2011^16^	★★★	★	★★	6	Low
Polan, 1980^23^	★	★	★	3	High
Puolakka, 1980^24^	★	★	★	3	High
Tjaden, 1990a^20^	★★★★	★★	★★★	9	Low
Tjaden, 1990b^20^	★★★	★	★★	6	Low
Zullo, 2003/2004^12,13^	★★★★	★★	★★★	9	Low

Selection comprised of representativeness of exposed cohort, selection of non-exposed cohort; ascertainment of exposure, and demonstration that outcome of interest was not present at start of study. Comparability comprised of study controls for baseline comorbidities and disease severity. Outcome comprised of assessment of outcome, was follow-up long enough for outcomes to occur, and adequacy of follow-up of cohorts. Studies classified as high (1–3 stars), intermediate (4–5 stars), or low (6–9 stars) risk of bias.

**Table 4:** Definitions of Key Study Design Elements in Controlled Studies of Conservative Surgery With or Without Presacral Neurectomy.

Study	Key Patient Selection Criteria	Procedural Details	Treatment Failure Definitions
Candiani, 1992^19^	• Laparotomic/Laparoscopic diagnosis of endometriosis stage III or IV Moderate or severe midline or midline plus lateral menstrual pelvic pain No previous gynecological surgery or medical treatment within 6 months	• Laparotomic conservative surgery as described by Buttram and Reiter^25^ PN as described by Malinak^26^ All adhesions & ovarian/peritoneal endometriotic implants removed during surgery	Recurrence of moderate or severe dysmenorrhea based on a 0–7 multidimensional pain scale where moderate was defined as a score of 4–5 and severe was 6–7.
Garcia, 1977^22^	• Endoscopic & histological diagnosis of endometriosis, 96% with stage III or IV disease	• Surgical excision of adhesions & endometriomas PN: no details provided Additional procedures performed in 13% of patients	Severe dysmenorrhea unchanged after surgery
Liu, 2011^16^	• Laparoscopic or histological diagnosis of endometriosis Secondary progressive dysmenorrhea symptoms Fertility preservation desires No planned additional procedures	• Conventional laparoscopic resection of endometriosis lesions, including cystectomy for ovarian endometrioma, unilateral uterine adnexectomy, and electrocautery for lesions of the pelvic peritoneum, and pelvic adhesiolysis PN: Longitudinal 2–3 cm incision of the posterior peritoneum at the anterior sacrum; presacral nerve trunk (mostly to left side) resected 1–2 cm	Less than 50% relief from severe dysmenorrhea after surgery
Polan, 1980^23^	• Chief complaint of chronic pelvic pain, all with infertility and some with endometriosis diagnosed by laparoscopy	• Lysis of adhesions & fulguration of endometriosis foci PN as described by Malinak^26^; 2–4 cm resection of neural tissue Additional procedures performed in an unspecified number of patients	Absence of pelvic pain relief after surgery
Puolakka, 1980^24^	• Endometriosis, mostly with dysmenorrhea (92%) or low back pain (82%)	• Laparoscopic or laparotomic excision of endometriosis and resection of ovaries and uterosacral ligaments PN: no details provided	No relief or deterioration in symptoms after surgery
Tjaden, 1990a^20^	• Endometriosis stage III/IV Moderate to severe midline dysmenorrhea	• Laparotomic conservative resection PN: Methods according to Rock and Jones^27^, and Rosenhein^28^	Absence of dysmenorrheic pain relief
Tjaden, 1990b^20^	• Endometriosis stage III/IV Moderate to severe midline dysmenorrhea	• Laparotomic conservative resection PN: Methods according to Rock and Jones^27^, and Rosenhein^28^	Absence of dysmenorrheic pain relief
Zullo, 2003/2004^12,13^	• Severe midline dysmenorrhea for at least 6 months Unresponsive to medical treatment Clinical or ultrasonographic evidence of endometriosis	• Electrosurgical excision (primarily) or ablation of visible pelvic endometriotic lesions, enucleation of endometriomas, and lysis of pelvic adhesions PN: Simplified Perez technique^29^	Continuing dysmenorrheic symptoms

Procedural data were reported inconsistently and without sufficient detail to warrant meta-analysis. Generally, procedure time was slightly longer (median: 13 minutes; range: 3 to 23 minutes) with PN, blood loss was comparable (range: −4 to 14 cc greater with PN), and hospital stay was comparable (range: 0 to 11 hours shorter with PN).

**Figure 2:**
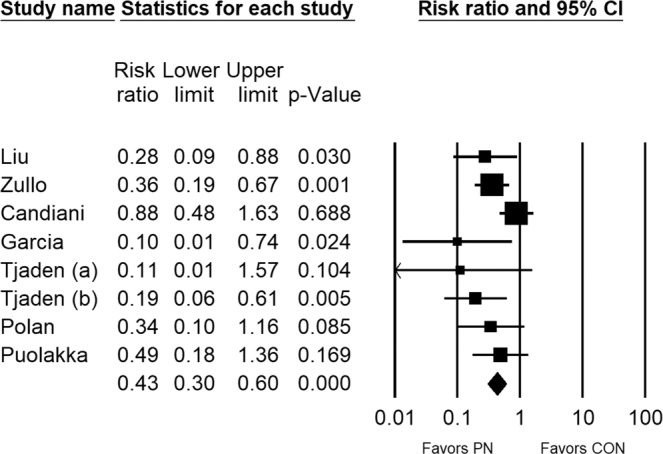
Forest plot of the risk of treatment failure comparing conservative surgery with or without presacral neurectomy. The risk ratio and 95% confidence interval are plotted for each study. The pooled risk ratio (diamond apex) and 95% confidence interval (diamond width) is calculated using a fixed effects model. Pooled risk ratio >1 suggests higher risk with presacral neurectomy. Pooled risk ratio <1 suggests lower risk with presacral neurectomy. Fixed effects risk ratio: 0.43 (95% CI: 0.30, 0.60; p < 0.001). I^2^ = 38%, p = 0.130. PN = presacral neurectomy; CON = Controls.

**Figure 3:**
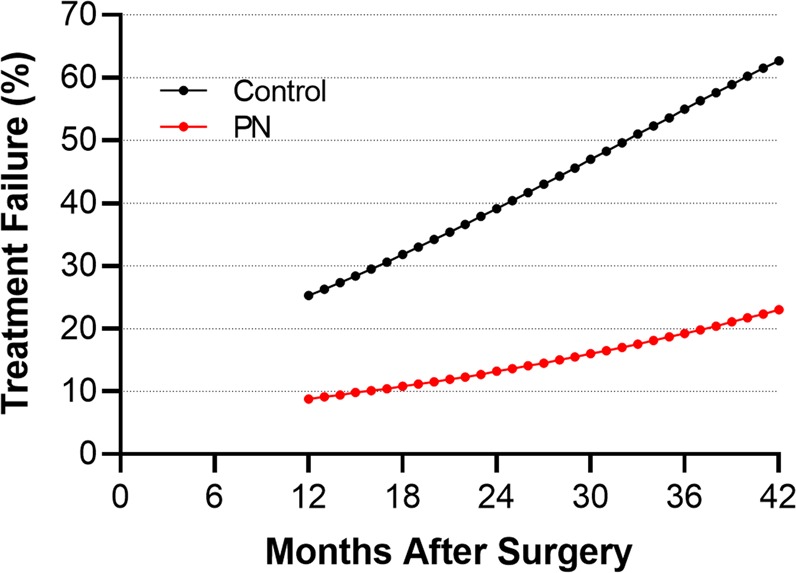
Meta-regression of the influence of follow-up duration on the risk of treatment failure comparing conservative surgery with or without presacral neurectomy. The risk of treatment failure at 1-year follow-up was 8.8% with conservative surgery and presacral neurectomy (PN) and 25.3% with conservative surgery alone (Control). Thereafter, the annualized risk of treatment failure was 5.9% per year with PN and 15.5% per year with Control (p = 0.034). Plotted values represent the regression line that spans the range of follow-up durations among studies included in the meta-analysis.

Among 8 group comparisons, crude rates of treatment failure were 15.0% (35/234) with PN and 40.9% (96/235) with Controls over 34 months median follow-up. Heterogeneity in the treatment failure rate among studies was small to moderate (I^2^ = 38%, p = 0.130). In fixed effects meta-analysis, the risk of treatment failure was lower with PN vs. Controls (RR = 0.43, 95% CI = 0.30 to 0.60, p < 0.001), representing a 57% risk reduction (Fig. 2). Funnel plot asymmetry was not evident and quantitative assessment did not indicate publication bias (p = 0.078). The treatment benefit of PN was maintained in a subgroup analysis that separately evaluated results among randomized (RR = 0.54, 95% CI = 0.35 to 0.84, p = 0.006) and nonrandomized (RR = 0.29, 95% CI = 0.17 to 0.50, p < 0.001) studies. In meta-regression, the benefit of PN persisted over a 42-month postoperative period. At 1-year follow-up, treatment failure rates were 8.8% with PN and 25.3% with Controls. Thereafter, annualized treatment failure rates were 5.9% per year with PN and 15.5% per year with Controls (p = 0.034) (Fig. 3). Results of the one-study removed analysis suggested that the meta-analysis conclusions were not significantly influenced by any single study. Specifically, the risk of treatment failure remained lower with PN vs. Controls following removal of each study one at a time from the meta-analysis, with the RRs ranging from 0.31 to 0.46 (all p < 0.001). Finally, the risk of treatment failure was comparable when applying a random effects meta-analysis model (RR = 0.37, 95% CI = 0.23 to 0.60, p < 0.001).

Among 6 group comparisons, crude rates of operative complications were 0.6% with PN and 0% with Controls (p = 0.498). In the only study in which an operative complication was reported^12,13^, a single patient treated with PN experienced significant bleeding from the middle sacral vein that was successfully managed with bipolar electrocauterization. Limited results were available for the remaining complications, which were each reported in only one or two studies. The risk of postoperative constipation was higher with PN vs. Controls (12.5% vs. 0%, p = 0.024). No treatment group differences were observed in the risk of reoperation (4.1% vs. 3.0%, p = 0.758) or urinary incontinence (5.0% vs. 0%, p = 0.195) (Table 5). Each patient with postoperative urinary incontinence was treated with antimuscarinics, which resulted in only slight symptom improvement. Heterogeneity was not observed for any reported complication (all I^2^ = 0%).

**Table 5:** Complication Rates in Controlled Studies of Conservative Surgery With or Without Presacral Neurectomy.

Complication	Studies	Complication Rate		Effect Size		Heterogeneity (I2, p-value)
		Presacral Neurectomy	Control	Risk Ratio (95% CI)*	P-value	
Operative complication	6	0.6% (1/175)	0% (0/171)	3.00 (0.13, 72.2)	0.498	0%, p = 0.556
Constipation	2	12.5% (11/88)	0% (0/91)	10.62 (1.36, 82.8)	0.024	0%, p = 0.556
Reoperation	2	4.1% (4/98)	3.0% (3/99)	1.25 (0.31, 5.06)	0.758	0%, p = 0.532
Urinary incontinence	1	5.0% (3/60)	0% (0/60)	7.00 (0.37, 133)	0.195	0%, p > 0.999

*Risk ratio >1 indicates higher complication risk with presacral neurectomy; risk ratio <1 indicates lower complication risk with presacral neurectomy. All effect sizes derived from fixed-effects meta-analysis.

## Discussion

Recurrence of pelvic pain is common following conservative surgery for endometriosis^14,15^, a finding that was confirmed in this systematic review and meta-analysis. We found that adjunctive PN may provide greater relief from endometriosis-related midline pain and a similarly low rate of operative complications compared to conservative surgery alone but may increase the risk of constipation postoperatively.

The main finding of this review was that adjunctive PN reduced the risk of treatment failure relative to conservative surgery alone. This finding was upheld in subgroup and sensitivity analyses and was not influenced by significant heterogeneity or publication bias. A statistically significant reduction in the risk of treatment failure with PN was identified in only 4 of 8 comparisons, which was likely attributable to the small sample sizes of individual studies. After pooling results from all studies, there was a large and consistent treatment benefit attributable to PN such that the number of women needed to treat with PN in order to prevent one treatment failure (i.e. number needed to treat) was 4. That more than 50% of women treated with conservative surgery alone experienced pain recurrence by 3 years underscores the clinical importance of these results.

However, treatment efficacy with PN must be balanced against the possibility of complications. The risk of constipation was higher in women receiving PN (12.5% vs. 0%). Among women reporting constipation, 73% reported symptom resolution with medical treatment, while the remaining 27% reported persistent symptoms, representing 3.4% of all women treated with PN. While these results were only reported in 2 studies^12,13,16^, the finding of increased constipation risk with PN is in agreement with previous studies^17^. While urinary incontinence is another known risk of PN, we did not identify statistical differences between groups for this complication. However, this analysis was underpowered to detect clinically meaningful differences between groups since urinary incontinence was reported in only one study^12,13^. Therefore, while results of this review do not provide evidence that PN increases urinary incontinence risk, this possibility cannot be discounted given the lack of outcome reporting among included studies. Importantly, women with endometriosis often suffer from bowel- and bladder-related symptoms preoperatively and it is unclear whether the data reported here represent pre-existing or new diagnoses. Although the reported intra- and post-operative complication rate was limited, under-reporting cannot be excluded as inconsistent complication reporting is common in the surgical literature^18^.

It is important to compare the results of the current review with those of prior systematic reviews of PN. In a Cochrane review published in 2005, Proctor and colleagues^4^ included one randomized trial of conservative surgery with or without adjunctive PN^19^ in their meta-analysis. They excluded the study of Tjaden *et al*.^20^ due to small sample size, and the randomized trial of Zullo *et al*.^12,13^ was not yet published. In a 2007 systematic review with meta-analysis, Latthe *et al*.^5^ included the same three randomized trials as in the current review, but their analysis evaluated pain at distinct time points, none of which were reported in all three trials. Therefore, the current systematic review is the only report to pool pain data from all randomized trials. Further, we supplemented this evidence with data from five nonrandomized controlled studies. Lastly, we performed subgroup, sensitivity, and meta-regression analyses to explore the robustness of study conclusions and the influence of potential confounders. In this respect, the current research is the most comprehensive and contemporary systematic review on the clinical utility of PN in women with endometriosis.

Despite the benefit of PN observed in this study, it is important to recognize that this is a technically challenging procedure with attendant risks of significant bleeding from the adjacent venous plexus. The surgery must be performed carefully and meticulously in order to prevent injury to major vessels and the right ureter, which delineate the lateral border of the dissection. In the current study, significant operative bleeding was reported in 1 (0.6%) woman treated with PN, with no other reports of operative complications. The single complication, sacral hemorrhage, represents the most common complication of PN^17^. Surgical experience with PN among surgeons in the included studies was unclear, but it is plausible that the incremental pain reductions and low complication rates with PN in this meta-analysis may have been realized among experienced practitioners. Future studies aimed to quantify the learning curve associated with PN are warranted. It is also important to note that, despite surgeon experience, adjunctive PN may not be appropriate in all women. Appropriate patients are those who report midline pain of at least 6 months duration that is refractory to medical management. Midline pelvic pain is most responsive to uterine denervation procedures^21^ whereas women with primarily lateral component pain typically show little improvement^12,13^. Aside from patient selection factors, neuroanatomic variability of the presacral space and failure to completely transect all presacral nerves are potential causes of reduced efficacy. While pre-existing constipation or urinary dysfunction are not absolute contraindications to PN, women with these diagnoses should be carefully informed of the potential for exacerbation of symptoms following surgery. Ultimately, the decision to undergo PN should be made following patient-physician shared decision-making that carefully considers medical history, physician operative experience, complication risk, and future fertility desires.

This meta-analysis has certain limitations pertaining to the quality of studies available for analysis that may influence interpretation. Heterogeneity in study design and surgical techniques was observed, which may confound data interpretation. We were unable to determine the influence of surgical access (laparoscopy vs. laparotomy) in treatment outcomes due to the small number of studies available for analysis. Inclusion of data from nonrandomized studies may have introduced bias into the results. Indeed, most included studies were classified as intermediate or high risk of bias. However, study design differences did not impact overall conclusions since pain relief with PN persisted when analyzing only randomized trials. There was also considerable variation in the completeness of data reported among studies such that only weak conclusions can be derived from outcomes that were reported in few studies. Patient selection criteria and surgeon experience were under-reported and, therefore, generalizability of these results may be limited. Further, the bulk of the results of this review were derived from older studies that utilized laparotomy. Yet, contemporary surgical management of endometriosis-related pain involves laparoscopic techniques that allow a more radical excision of endometriotic lesions with better long-term outcomes. Despite the fact that scientific reports on PN have decreased over the last decade, PN is widely promoted by specialized endometriosis treatment centers as a relatively safe and efficacious adjunct to conservative surgery, a conclusion which is debatable based on numerous factors including surgeon experience and limited evidence derived from studies with unclear generalizability to current practice. It seems there may be a lack of concordance of the role of PN between scientific reports and in clinical practice at specialized centers where PN is aggressively promoted. Given the relative paucity of controlled studies on PN despite its continued use in clinical practice, additional randomized trials and high-quality nonrandomized studies with long-term follow-up are encouraged to better characterize the benefits and harms associated with this procedure.

## Conclusion

In well-selected patients, conservative surgery with adjunctive PN may provide greater relief from midline pain and a similarly low rate of operative complications relative to conservative surgery alone but may increase the risk of constipation postoperatively. Main limitations of this review included unclear generalizability of results due to under-reporting of patient selection criteria, surgeon experience, and complications, as well as relatively short follow-up duration in a young patient population. Further, results were derived from mainly older and lower quality studies. Since then, surgical techniques to treat endometriosis have been improved and the effect of PN observed in prior studies should be confirmed in future studies in women in whom radical excision of deep infiltrating lesions is obtained.

## Data Availability

The underlying data informing this meta-analysis will be made available upon reasonable request.
